# Visual Fixation Assessment in Patients with Disorders of Consciousness Based on Brain-Computer Interface

**DOI:** 10.1007/s12264-018-0257-z

**Published:** 2018-07-17

**Authors:** Jun Xiao, Jiahui Pan, Yanbin He, Qiuyou Xie, Tianyou Yu, Haiyun Huang, Wei Lv, Jiechun Zhang, Ronghao Yu, Yuanqing Li

**Affiliations:** 1 Center for Brain Computer Interfaces and Brain Information Processing, South China University of Technology, Guangzhou, 510640 China; 2 Coma Research Group, Centre for Hyperbaric Oxygen and Neurorehabilitation, General Hospital of Guangzhou Military Command, Guangzhou, 510010 China

**Keywords:** Visual fixation, Brain-computer interface, Disorder of consciousness, Coma recovery scale-revised, Electroencephalography

## Abstract

Visual fixation is an item in the visual function subscale of the Coma Recovery Scale-Revised (CRS-R). Sometimes clinicians using the behavioral scales find it difficult to detect because of the motor impairment in patients with disorders of consciousness (DOCs). Brain-computer interface (BCI) can be used to improve clinical assessment because it directly detects the brain response to an external stimulus in the absence of behavioral expression. In this study, we designed a BCI system to assist the visual fixation assessment of DOC patients. The results from 15 patients indicated that three showed visual fixation in both CRS-R and BCI assessments and one did not show such behavior in the CRS-R assessment but achieved significant online accuracy in the BCI assessment. The results revealed that electroencephalography-based BCI can detect the brain response for visual fixation. Therefore, the proposed BCI may provide a promising method for assisting behavioral assessment using the CRS-R.

## Introduction

Patients with severe brain injury may suffer from disorders of consciousness (DOCs), including coma, vegetative state (VS), and minimally conscious state (MCS). Accurate assessment of the status of DOC patients is important for monitoring the effects of treatment and the rehabilitation process. The proposed behavioral scales for DOC patients include the Coma Recovery Scale-revised (CRS-R), the Glasgow Coma Scale, the Full Outline of Unresponsiveness, the Wessex Head Injury Matrix, and the Sensory Modalities Assessment and Rehabilitation Technique. Among them, the CRS-R provides a more fine-grained assessment and has demonstrated significant sensitivity in differentiating MCS from VS patients [1]. The CRS-R contains 6 subscales addressing auditory, visual, motor, oromotor, communication, and arousal processes [1]. Each subscale consists of several items that are critical for identifying subtle behavioral markers of recovery of consciousness, and there are a total of 23 hierarchically-arranged items. The visual subscale includes five items: visual startle, visual fixation, visual pursuit, object localization, and object recognition, corresponding to scores of 1, 2, 3, 4, and 5, respectively [2–4].

Visual fixation plays a key role in the differentiation between VS and MCS, and there are many behavioral scales available for its assessment in patients with DOCs. Sustained visual fixation is considered as present when the eyes change from an initial fixation point and re-fixate on a new target location for > 2 s in the CRS-R criteria. However, it is still a matter of debate whether visual fixation indicates automatic subcortical processing or higher-order cortical processing that heralds consciousness. And the different stimuli used in different behavioral scales may affect the accuracy of the diagnosis. Therefore, visual fixation assessment has been intensively studied in DOC patients [5–7]. Bruno *et al.* studied cerebral metabolism in ten DOC patients, five in a VS without fixation and five presenting visual fixation but otherwise all criteria typical of the VS. Patients without fixation showed metabolic dysfunction in a widespread fronto-parietal cortical network and this did not differ from the brain function seen in patients with visual fixation. Recovery rates did not differ between patients without or with fixation. Their findings suggested that sustained visual fixation in DOC patients does not necessarily reflect consciousness and higher-order cortical brain function [5]. Di *et al.* assessed visual fixation in 81 MCS or VS patients, and the occurrence of fixation to different stimuli was analyzed with the χ^2^ test. The results showed that 40 (49%) of the 81 patients showed fixation to visual stimuli. Among these, significantly more patients (39, 48%) showed visual fixation elicited by a mirror compared to a ball (23, 28%) and a light (20, 25%). Therefore, they concluded that using a mirror to test visual fixation is a sensitive and accurate method [6]. Naro *et al.* evaluated the visuomotor integration (VMI) and visual P300 in DOC patients in order to detect residual visuomotor network functionality that potentially sustains aware visual fixation. They found that the MCS patients showed preserved patterns of VMI and P300, whereas nearly all VS patients had no significant VMI. Nonetheless, two fixating VS individuals had a VMI similar to MCS patients. Their data suggest that some VS patients showing visual fixation can be aware [7]. However, the assessment of visual fixation is still a challenging task. First, DOC patients are severely lack of behavioral responses to external stimuli because of motor impairment, such that unambiguous signs of fixation can be difficult to observe or are easily missed [8, 9]. Second, visual responsive behavior for different stimuli has been shown to differ greatly [6]. Moreover, there are problems of subjectivity and misinterpretation of the behavioral response on the part of examiners [10]. Therefore, the challenge of behaviorally assessing visual fixation may result in frequent misdiagnosis in DOC patients as reported in previous studies [11–14].

To overcome the above limitations, brain-computer interfaces (BCIs), which directly detect the endogenous brain responses to external stimuli in the absence of behavioral expression, may provide a solution to improve clinical assessment and complement classical behavioral observations [9]. Our previous studies applied BCIs to assist the assessment of two items in the CRS-R scale: auditory startle and communication [15, 16]. To further expand that study, here we propose a visual BCI system to detect visual fixation in DOC patients.

## Materials and Methods

### Participants

Five healthy participants (four males; 29 ± 5 years old) first participated in the BCI experiments to validate the proposed BCI system. Next, 15 DOC patients (13 males and 2 females, 13–73 years old) from the General Hospital of Guangzhou Military Command were assessed by BCI and CRS-R (Table 1), and the clinical diagnosis was made according to the CRS-R. The CRS-R evaluation was performed one week before the BCI experiment. Each evaluation consisted of five repeated CRS-R assessments on separate days of a week. For each patient, the CRS-R scores were based on his/her best responses in the repeated CRS-R assessments. The CRS-R scores are not given for patient 15 because he had locked-in syndrome.

**Table 1:** Summary of clinical status of patients.

Patient index	Age	Gender	Etiology	Time since insult (months)	Clinical diagnosis	CRS-R score (auditory-visual-motor-oromotor-communication-arousal)
Patient 1	72	Male	NTBI	4	VS	4 (1-0-1-0-0-2)
Patient 2	16	Male	TBI	2	VS	5 (1-0-2-0-0-2)
Patient 3	52	Male	NTBI	6	VS	5 (1-1-0-1-0-2)
Patient 4	35	Female	NTBI	3	VS	5 (1-0-1-1-0-2)
Patient 5	40	Male	NTBI	3	VS	6 (1-1-1-1-0-2)
Patient 6	33	Male	TBI	5	VS	7 (1-1-2-1-0-2)
Patient 7	32	Male	NTBI	3	VS	7 (1-0-2-2-0-2)
Patient 8	52	Male	NTBI	4	VS	7 (1-1-2-1-0-2)
Patient 9	20	Male	NTBI	6	MCS	9 (1-1-4-1-0-2)
Patient 10	13	Male	TBI	2	MCS	9 (1-1-4-1-0-2)
Patient 11	45	Male	NTBI	1	MCS	10 (1-3-3-1-0-2)
Patient 12	69	Female	NTBI	10	MCS	11 (1-3-5-0-0-2)
Patient 13	61	Male	TBI	4	MCS	15 (2-5-5-1-0-2)
Patient 14	42	Male	NTBI	6	EMCS	20 (4-5-6-1-2-2)
Patient 15	73	Male	NTBI	12	LIS	–

LIS, locked-in syndrome; MCS, minimally-conscious state; EMCS, emerging from MCS; NTBI, non-traumatic brain injury; TBI, traumatic brain injury; VS, vegetative state. In the CRS-R score column, the second numbers in brackets are the sub-scores of visual function. A value of 2 in the second sub-score means that the patient showed visual fixation in the behavioral assessment.

The experimental protocols for the DOC patients were approved by the Ethics Committee of the General Hospital of Guangzhou Military Command, and complied with the Code of Ethics of the World Medical Association (Declaration of Helsinki). Written informed consent and authorization to publish individual details in this manuscript were obtained from the healthy participants and the patients’ legal surrogates.

### Data Acquisition

A SynAmps^2^ amplifier (Neuroscan Compumedics, El Paso, TX) was used to record scalp EEG signals at a sampling rate of 250 Hz and the signals were filtered with a bandpass filter between 0.05 Hz and 100 Hz. A 32-channel EEG cap (LT37) that followed the standard 10–20 system was worn by each participant and the EEG signals were referenced to the right mastoid. The impedances of all electrodes were < 5 kΩ during data acquisition.

### Graphical User Interface and Experimental Paradigms

**Fig. 1:**
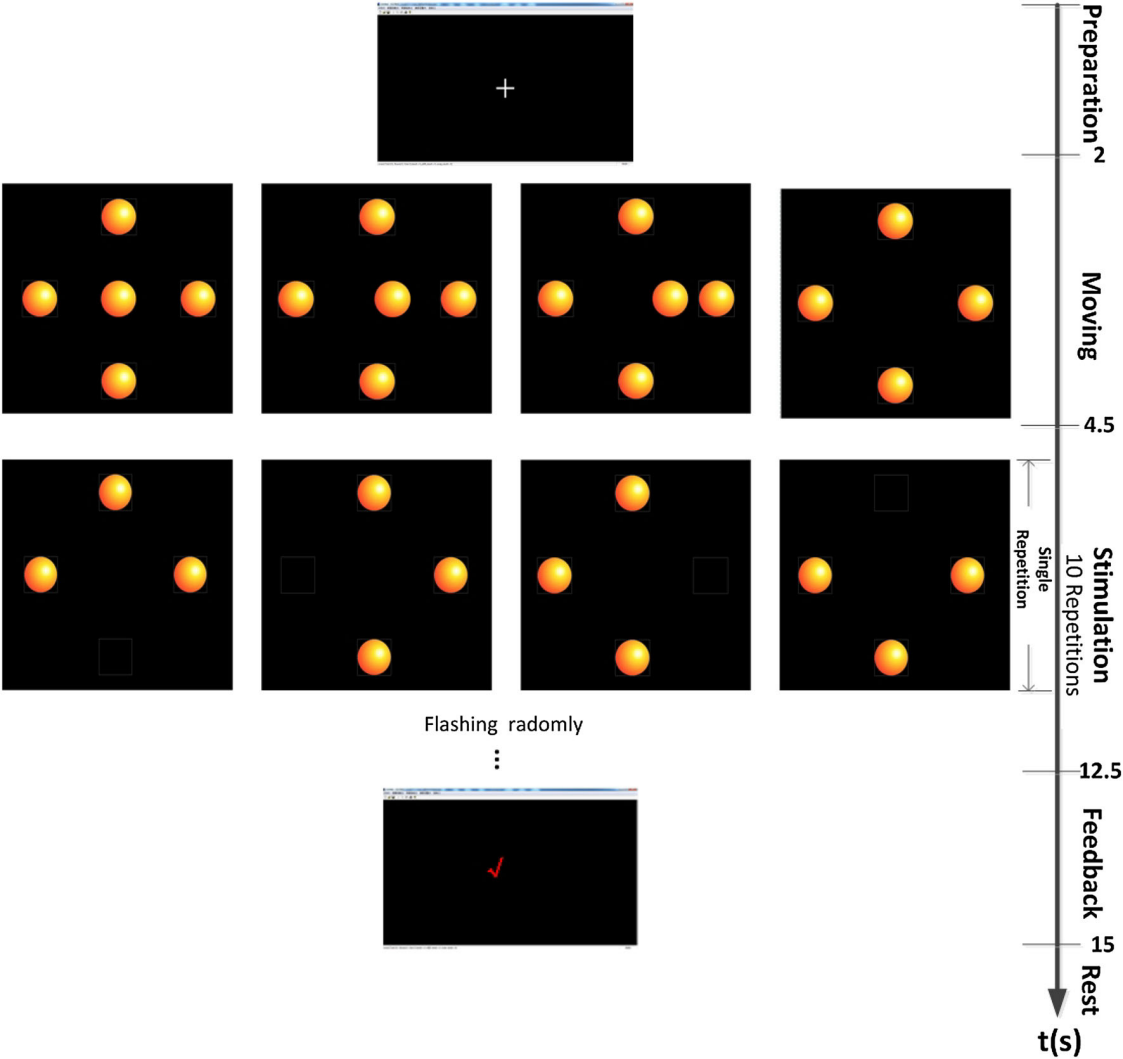
The GUI and the paradigm of the experiment. A square area was set and four brightly-colored ball buttons were arranged at the edge of the square in four locations. A trial was started with “+” and a ball in the middle of the GUI moved to guide the patient to the new target location that was chosen randomly from the four directions. When the ball overlapped with the target button, the four buttons flashed randomly. The stimulation contained 10 repetitions of the flash.

Each participant was seated in a comfortable chair (a wheelchair for patients)  0.5 m from the monitor, which was 27.38 cm high and 48.7 cm wide. The graphical user interface (GUI) consisted of a square in the center of the screen (Fig. 1). The side length of the square was 27.38 cm (corresponding to a visual angle of 28.7°), equal to the height of the monitor. In this area, four buttons were arranged at the edge of the square in four locations: left, right, above and below. In the CRS-R-based behavioral assessment, the clinician moved a mirror to one of four locations (right, left, above, and below) in a plus-shaped tracking pattern. Therefore, four buttons rather than some other number were designed into the GUI to keep the BCI assessment as close as possible to the CRS-R-based behavioral assessment. One of the four buttons was pseudo-randomly chosen as a target location, and then a button appeared in the center of the square and moved to the target location to guide the patient to re-fixate the new target button. The foreground of all buttons was presented as pictures of a brightly-colored ball, while the background was traditional black. All pictures were cropped to squares according to the full size of the monitor screen, e.g., 4 ×  4 cm^2^ in this study.

The paradigm of the proposed BCI system simulated the behavioral assessment of visual fixation in the CRS-R, in which the patient’s eyes change from an initial point to a new target location and re-fixate on the new location for > 2 s. As shown in Fig. 1, each experimental trial began with the visual prompt of a cross lasting 2 s, a target location was randomly chosen from the four directions, and a brightly-colored ball appeared in the middle of the GUI to guide the patient. Then the middle ball moved from the center to the target location, while the four buttons at the edge of the GUI did not move. The movement lasted 2.5 s and the distance between the center point of the button’s initial location and the center point of the new target location was 11.5 cm (corresponding to a visual angle of 13.1°). After the moving ball reached the target location and overlapped with the corresponding button, the four buttons flashed from the foreground to the background in random order, the background appearing for 100 ms at an inter-stimulus interval of 100 ms. In the stimulation round, each button flashed once. Each experimental trial included ten repetitions of stimulation. When the ten repetitions were finished (8 s), the BCI algorithm used the EEG data to determine the button on which the subject re-fixated. If the target was detected, which implied that the target was correctly followed and re-fixated, a tick was presented for 2.5 s in the center of the GUI. However, no feedback was given for an incorrect result. The time course of a trial is illustrated in Fig. 1. After the trial finished, there was a break of 1–10 s before the next trial began. During the experiment, the patient was verbally encouraged to fixate on the moving target and to avoid decreased arousal as assessed by the clinician.

### Experimental Procedures

First, five healthy individuals participated in the BCI experiment to validate the proposed system. Each participant completed two sessions of the BCI experiment. Each session consisted of two blocks, a calibration block of 10 trials and an online block of 10 trials.

Subsequently, 15 DOC patients participated in two different visual fixation assessments: CRS-R-based behavioral assessment and BCI-based assessment. (1) The CRS-R assessment was conducted by an experienced clinician. Following the standard protocol, the clinician held a brightly-colored ball 15–20 cm in front of the patient’s face and then rapidly moved to the upper, lower, right, and left visual fields for a total of 4 trials. The criterion was that the patient’s eye changed from the initial fixation point and re-fixated on the new target location for > 2 s. If at least 2 episodes of fixation were achieved by the patient, he/she was scored 2 for the visual fixation item, otherwise, the lower item of visual startle (item score of 1) was given. (2) The BCI-based assessment was conducted for the 15 patients using the same BCI system as for the healthy participants. Each patient also completed two sessions of the BCI experiment as described above.

### EEG Data Analysis

Different event-related potential (ERP) components are often used by BCI algorithms to select a target from several interferences, i.e., the other three non-target buttons. According to previous studies [17, 18], ERP features can be constructed from filtered EEG signals, and linear or nonlinear classifiers can be used to determine whether the target is fixated.

The general ERP-based BCI paradigm includes one target and *K*–1 interferences (*K* stimuli in total). $X = (X^{(1)} , \ldots ,X^{(K)} )$ denotes the EEG data epoch after stimulus onset. In $X^{(l)} \in R^{C \times T}$, C and T indicate the channels and time points respectively. If $a \in (1, \ldots ,K)$ is the real target on which the participant fixates, identifying the target from the interferences can be described as follows:

1 $$ f_{\theta } (X^{(l)} ) = \left\langle {W,X^{(l)} } \right\rangle + b $$

2 $$ p_{\theta } (a\left| X \right.) = \frac{{e^{{f_{\theta } (X^{(a)} )}} }}{{\sum\nolimits_{l = 1}^{K} {e^{{f_{\theta } (X^{(l)} )}} } }} $$

where $p_{\theta } (a\left| X \right.)$ is the probability of the related ERP response after each stimulus. To predict the target, we maximized the posterior probability $p(a\left| X \right.)$ given *X* with respect to *α*, then the target is the maximum output of the model $f_{\theta } (X^{(l)} )$ as follows:

3 $$ \hat{a} = \text{arc} \mathop {\hbox{max} }\limits_{a} \log p_{\theta } (a\left| X \right.) = \text{arc} \mathop {\hbox{max} }\limits_{a} f_{\theta } (X^{(a)} ) $$

In our study, the collected EEG signals for all trials were band-pass filtered between 0.5 and 20 Hz. The data epochs corresponding to each stimulus were extracted from 0 to 600 ms after stimulus onset. All epochs were re-referenced to the mean amplitude in the baseline, 100 to 0 ms before stimulus onset. We down-sampled the data epoch by a rate of 6 in each channel and the down-sampled epoch contained 25 data points. A feature vector $X^{(l)}$, with a length of 750 (30 channel × 25 data points), was constructed by concatenating the data points of each channel. The vector $w$ was re-shaped from the coefficient matrix $W$ using a similar process. Therefore, the detection model becomes:

4 $$ f(X^{(l)} ) = w^{T} X^{(l)} + b $$

5 $$ \hat{a} = \text{arc} \mathop {\hbox{max} }\limits_{a} f(X^{(a)} ) $$

A linear Support Vector Machine classifier was used to solve this model with training data. For the online test, the model (Eq. 4) was applied to predict the target stimulus that corresponded to a high score. Finally, the ratio of the number of trials with correct responses to the total number of trials in online blocks was calculated as the online accuracy of the BCI assessment

### Statistical Analysis

To obtain the significance level of the accuracy, the χ^2^ statistical test was used. Specifically, the χ^2^ statistic was calculated as follows [19]:

6 $$ \chi^{2} = \sum\limits_{i = 1}^{k} {\frac{{(f_{oi} - f_{ei} )^{2} }}{{f_{ei} }}} $$

where $f_{oi}$ and $f_{ei}$ are the observed and expected frequencies of the *i*th class (*i* = 1, 2,…,*k*). In this study, a target was determined from the four flashing buttons. Therefore, the chance level that the target was selected was 0.25, whereas the chance level for selecting a non-target was 0.75. Considering that 20 trials of the online BCI test were conducted for each participant, the expected $f_{e1}$ was 5 and the expected $f_{e2}$ was 15. Furthermore, $f_{o1}$ and $f_{o2}$ were the numbers of times that the target (*i* = 1) or a non-target (*i* = 2) was determined in the online BCI test. Using a significance level of *P* = 0.05, we obtained a value of 3.84 for the *χ*^2^ test (degrees of freedom: 1), which corresponded to 9 hits in 20 trials or an accuracy of 45%. Therefore, a participant was considered visual fixation responsive in the BCI assessment if he/she obtained a significant BCI accuracy.

## Results

### Results for Healthy Participants

The averaged online accuracy of BCI assessment in the healthy group was 84% ± 3.4%. Furthermore, each healthy participant achieved a significant accuracy (significance level: 45%, *P *< 0.001, *χ*^2^-test), as detailed in Table 2. The effectiveness of our BCI system for visual fixation detection was thus demonstrated.

**Table 2:** Online results of healthy participants.

Healthy participants	Number of trials	BCI online accuracy (%)	*P* value (*χ*^2^-test)
HC1	20	80	0.0146
HC2	20	85	0.0035
HC3	20	85	0.0035
HC4	20	90	< 0.001
HC5	20	80	0.0146
Mean ± SD	20	84 ± 3.4	< 0.001

**Fig. 2:**
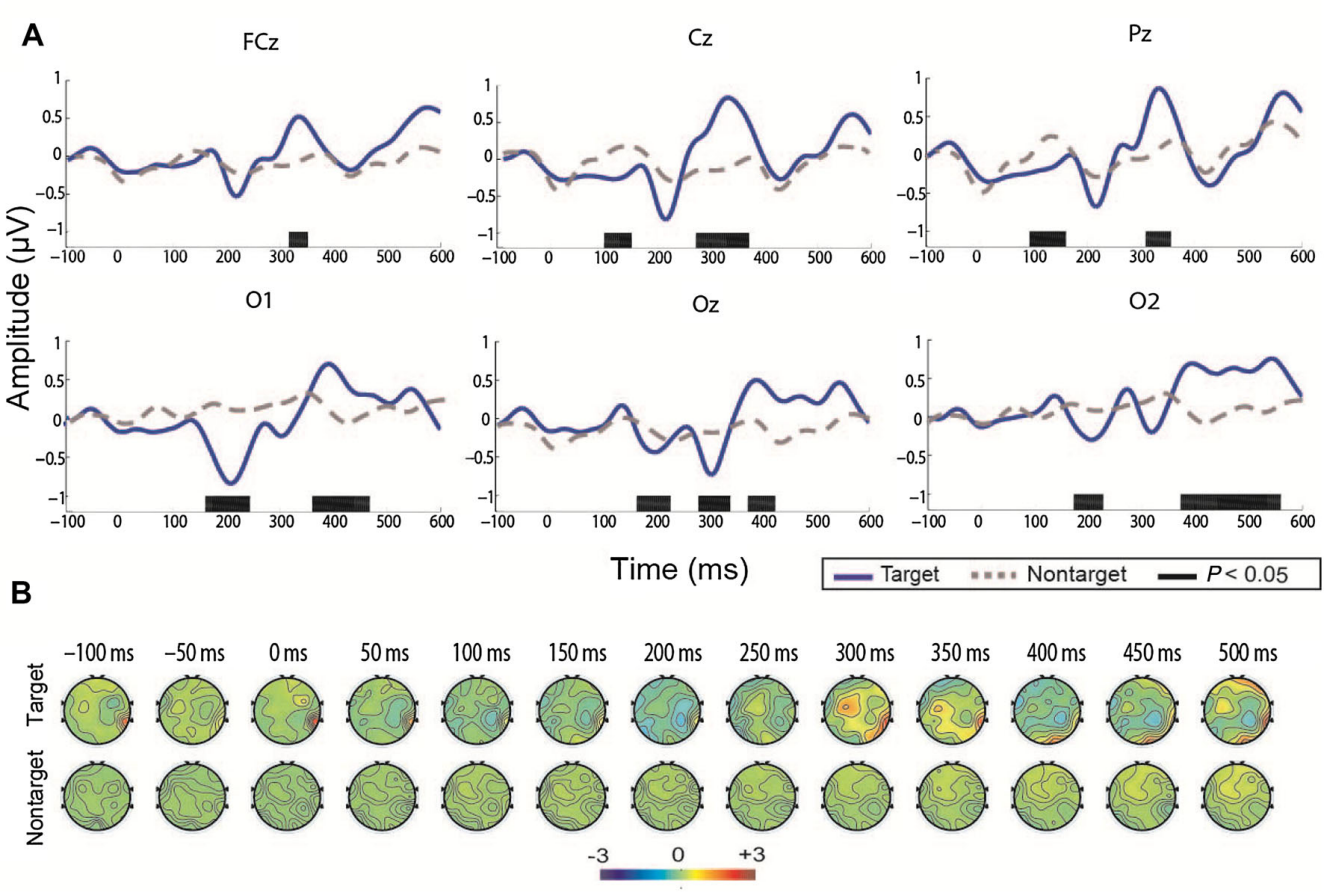
Averaged ERP waveforms and scalp topography of five healthy participants. **A** Waveforms from 100 ms before the stimulus onset to 600 ms after stimulus onset in the six channels (FCz, Cz, Pz, O1, Oz, and O2). Black bars, time intervals with significant differences of the waveforms evoked by target and non-target (two-sample *t*-test, *P *< 0.05). **B** Scalp topography averaged from the 5 healthy participants.

Offline ERP analysis was performed to further demonstrate the physiological plausibility of the proposed BCI system. We calculated the temporal ERP waveforms by averaging the 40 trials of EEG epochs for each participant. The group-averaged ERP waveforms were also obtained by averaging them across all 5 participants. The group-averaged ERP waveforms of healthy participants from FCz, Cz, Pz, O1, Oz, and O2 are shown in Fig. 2. We first found a positive component between 300 and 400 ms in FCz, Cz, and Pz, and a broad positive component between 350 ms and 600 ms in O1, Oz, and O2, which was associated with P300. There was a negative component at 200 ms defined as the motion-specific visual evoked potential N200 in the six selected channels. The point-wise running *t*-test was also used to compare the difference between the responses elicited by target and non-target, and multiple comparison correction with a cluster size of 7 was used [20–22]. There were remarkable differences between the responses elicited by the target and non-target stimuli in the period 300–400 ms in FCz, Cz, and Pz, and in the periods 200–300 ms and 350–500 ms in O1, Oz, and O2 (Fig. 2A). The N200 and P300 components were clearly elicited by the target stimuli. The N200 occurred slightly more than 200 ms after stimulus onset and was significant in O1, Oz, and O2. The P300 with a latency between 300 and 500 ms was significant in the six channels and was broad in O1, Oz, and O2. Furthermore, no ERP component was elicited by the non-target stimuli, and this result coincided with the physiological principles in reported references [23–27]. The averaged scalp topography of the 5 healthy participants is also shown in Fig. 2B. We found that the negative component N200 was distributed in the temporo-occipital and associated parietal areas between 200 and 250 ms. The P300 was distributed in the central regions between 300 and 350 ms. The online accuracy rates, the apparent ERP waveforms, and reasonable distribution in scalp topography of the healthy participants validated the effectiveness of the BCI paradigm.

### Results from Patients

The 15 DOC patients participated in the BCI experiment over 7 months; their online results are shown in Table 3. In the BCI assessment, an online accuracy > 45% was considered to show visual fixation. In the CRS-R assessment, a visual subscale score (the second sub-score in the CRS-R scores) of 2 means that visual fixation behavior was detected by the clinician.

**Table 3:** BCI online and CRS-R results for DOC patients.

Patient	Trials	Hits	BCI online accuracy (%)	*P* value	CRS-R score
Patient 1	20	4	20	0.61	4 (1-0-1-0-0-2)
Patient 2	20	5	25	1	5 (1-0-2-0-0-2)
Patient 3	20	6	30	1	5 (1-1-0-1-0-2)
Patient 4	20	4	20	0.61	5 (1-0-1-1-0-2)
Patient 5	20	5	25	1	6 (1-1-1-1-0-2)
Patient 6	20	8	40	0.24	7 (1-1-2-1-0-2)
Patient 7	20	4	20	0.61	7 (1-0-2-2-0-2)
Patient 8	20	11	**55**	0.0019	7 (1-1-2-1-0-2)
Patient 9	20	8	40	0.24	9 (1-1-4-1-0-2)
Patient 10	20	7	35	0.61	9 (1-1-4-1-0-2)
Patient 11	20	5	25	1	10 (1-3-3-1-0-2)
Patient 12	20	9	**45**	0.04	11 (1-3-5-0-0-2)
Patient 13	20	9	**45**	0.04	15 (2-5-5-1-0-2)
Patient 14	20	7	35	0.61	20 (4-5-6-1-2-2)
Patient 15	20	13	**65**	< 0.001	locked-in syndrome

Accuracies > 45% (*P* = 0.05, *χ*^2^ test) are marked in bold.

First, among the 15 patients, patients 12, 13, and 15 achieved significant accuracy and were considered to be responsive to visual fixation in the BCI assessment. The visual subscale scores were 3 for patient 12 and 5 for patient 13, indicating that higher visual behavior was detected by CRS-R. Patient 15 (locked-in syndrome) had recovered consciousness and showed consistent command-following. Thus, the three patients were classified into the responsive group by both BCI and CRS-R. Second, 9 of the 15 patients (patients 1, 2, 3, 4, 5, 6, 7, 9, and 10), who did not show visual fixation in either the BCI or the CRS-R assessment, were classified into the non-responsive group. In this group, patients 1, 2, 4, and 7 failed to show visual fixation. Clinical assessment showed that these four patients failed to show eye-blinks in response to threat and their visual subscale scores were 0 (Table 3), indicating impaired brainstem reflexes. Patients 3, 5, 6, 9, and 10 had a visual subscale score of 1, which implied that visual fixation was not detected by the CRS-R behavioral scale. Third, patients 11 and 14 did not achieve significant accuracy in the BCI assessment but showed higher visual behavior (patient 11) or cognitive behavior (patient 14) in CRS-R assessment. Patient 11 showed visual pursuit behavior, a higher behavior item than visual fixation in CRS-R. Patient 14 showed higher cognitive functions and was considered conscious in the clinical assessment. However, their BCI online accuracy was close to the chance level of 25%. So patients 11 and 14 were classified into the non-responsive group by BCI assessment (BCI-non-responsive group). Finally and more importantly, patient 8 showed no visual fixation in the CRS-R measurement before the BCI measurement, but achieved significant online accuracy in the BCI assessment (Table 3) and was classified into the responsive group by BCI (BCI-responsive group).

**Fig. 3:**
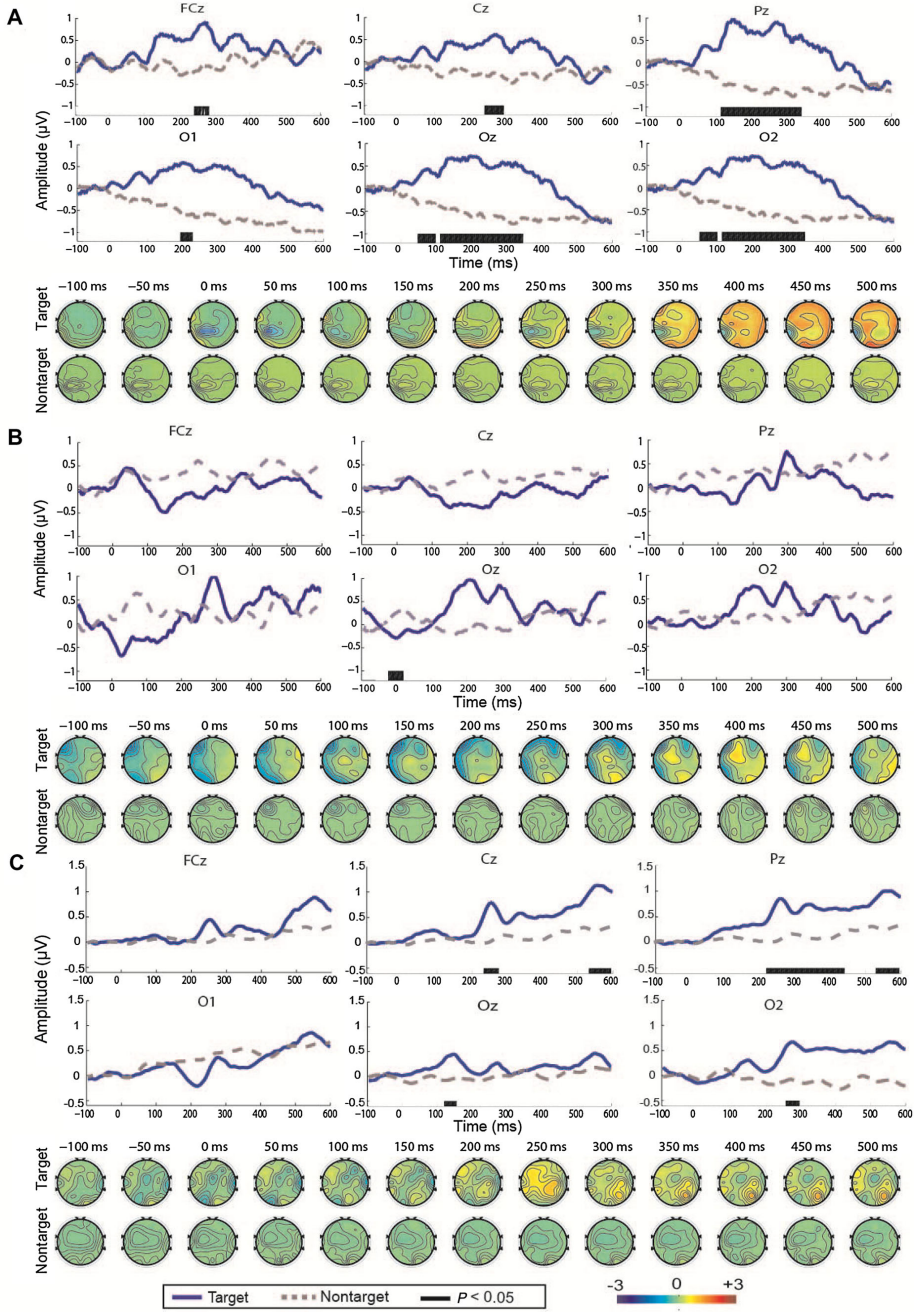
**A**–**C** ERP waveforms and scalp topography in patients 12 (**A**), 13 (**B**), and 15 (**C**). The waveforms were obtained from 100 ms before the stimulus onset to 600 ms after the stimulus onset in six channels (FCz, Cz, Pz, O1, Oz, and O2). The blue solid and gray dashed lines denote the waveforms evoked by the target and non-target stimuli, respectively.

In patients 12, 13, and 15 in the responsive group, the averaged ERP waveforms and scalp topography were also obtained by averaging all 40 trials for each patient (Fig. 3). In patient 12, a broad positive component related to P300 was exhibited in all six channels, and there were significant differences between the waveforms elicited by the target and non-target stimuli in the period from 200 ms to 300 ms in FCz, Cz, and O1 and in the period from 100 ms to 400 ms in Pz, Oz, and O2 (Fig. 3A). In addition, positive components were found over the whole scalp between 350 ms and 500 ms (Fig. 3A, lower panels). In patient 13, there were negative components in FCz and Cz, but a tiny positive peak occurred at 300 ms in Pz, O1, Oz, and O2. Remarkable differences between the responses elicited by the target and non-target stimuli were not observed throughout the period from 100 ms to 600 ms, except at 0 ms in Oz (Fig. 3B). The scalp map showed a negative component distributed in the lateral temporal and central areas. After 300 ms, a positive component was generated from the central and associated occipital sites (Fig. 3B, lower panels). In the locked-in syndrome patient 15, there was a significant positive peak in the range 200–300 ms in Cz and O1, and a broad positive component between 200 ms and 600 ms in Pz, especially in the periods 200–400 ms and 500–600 ms (Fig. 3C). The ERP component associated with P300 emerged between 300 and 500 ms and was distributed in the centro-parietal regions (Fig. 3C, lower panels). These findings are consistent with the neurophysiological results reported in previous publications [27–30].

However, the patients in the non-responsive group and BCI-nonresponsive group did not have any visual fixation response in the BCI assessment. Moreover, there was no corresponding ERP component in these patients’ individual ERP waveforms. The clinical results from patients in the non-responsive group were consistent with the BCI results. The patients in the BCI-nonresponsive group, including patients 11 and 14, showed higher visual function but no visual fixation by BCI assessment.

**Fig. 4:**
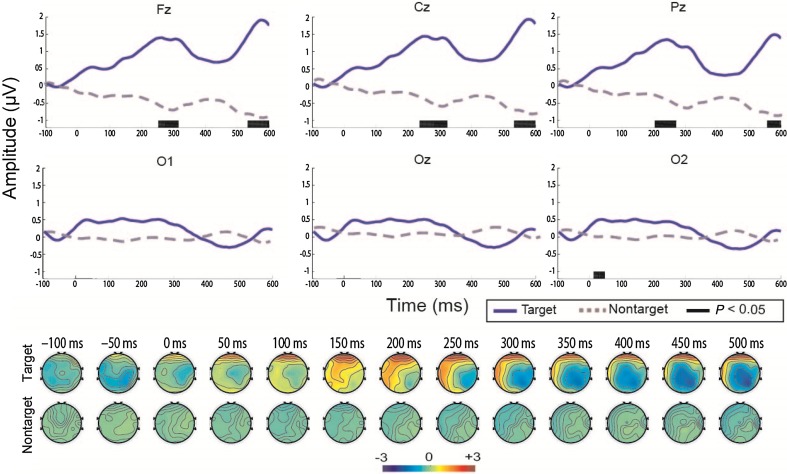
ERP waveforms and scalp topography in patient 8. The waveforms were obtained from 100 ms before the stimulus onset to 600 ms after the stimulus onset in the six channels (FCz, Cz, Pz, O1, Oz, and O2). The blue solid and gray dashed lines denote the waveforms evoked by the target and non-target stimuli, respectively.

Interestingly, patient 8 showed no visual fixation behavior in the CRS-R measurement but achieved significant online accuracy in the BCI assessment (Table 3). He showed a significant positive peak in the range 200–400 ms in FCz, Cz, and Pz, distributed in the frontal-central, central, and related parietal regions, and a negative component after 350 ms in the occipital area (Fig. 4).

## Discussion

In this study, a visual BCI system was developed to assist visual fixation assessment in patients with DOCs. Specifically, this BCI system was designed to simulate the behavioral evaluation of visual fixation in the CRS-R, in which a button (similar to the moving object in the behavioral assessment) was presented in the center of the GUI and moved to a new target position. The moving button aided patients to re-fixate on the target position. The patients were instructed to follow the moving button and re-fixate on the new position while their EEG signals were collected. Based on the EEG data, the BCI algorithm determined whether the patient re-fixated on the target. The efficacy of the proposed system was first demonstrated by an experiment involving five healthy participants. Then, 15 patients with DOCs participated in the assessments based on the BCI system and the CRS-R. Among the 15 patients, three who exhibited higher visual function in the CRS-R assessment were also found to be visual fixation responsive in the BCI assessment. The corresponding ERP components were evident in their ERP waveforms. However, 11 out of 15 patients did not exhibit visual fixation in the BCI assessment. Specifically, 2 of 11 patients exhibited visual pursuit and command following clinical assessment. Nine of the eleven patients did not show visual fixation behavior in CRS-R behavioral assessment. More importantly, one patient did not show visual fixation behavior in CRS-R but exhibited visual fixation in BCI measurement.

Motor disability poses a practical challenge for clinicians working with DOC patients in terms of diagnosis, care, and rehabilitation [31–33]. In clinical assessment, the behavioral scale, which remains the traditional way to evaluate consciousness in these patients, is highly dependent on motor abilities. The BCI may provide a method to directly detect the endogenous brain response of DOC patients to external stimuli, independent of behavioral expression. Recently, the “command-following” or “communication” BCI paradigm has helped to reduce the possibility of misdiagnosis of those DOC patients who have severely impaired motor abilities [31, 34, 35]. However, this type of protocol still requires high-level cognitive abilities, such as language understanding and task switching, compared to behavioral assessment. Therefore, poor performance and a high rate of false-negatives (patients showing command-following at the beside but not detected with BCI; 22%–94%) [36] are well-known limitations of BCIs for detecting command-following and communication in DOC patients. This issue highlights the current need to develop more reliable tools for the diagnosis of patients with DOCs. This study was designed to explore the feasibility of using visual BCI systems to complement the visual fixation behavioral assessments. The proposed BCI system was designed to simulate the behavioral visual fixation evaluation in CRS-R, the four buttons in different locations flashed and elicited the time-locked features of ERP components. The combination of CRS-R and BCI assessment may help to provide more precise, objective, and sensitive diagnoses. In this manner, the BCI’s poor performance can be improved because our system did not require that the patient have much more capacity than required by the current BCI for detecting command-following and communication.

For the proposed novel BCI paradigm, the motion of stimuli embedded into onscreen virtual buttons and the rare but task-relevant flashes have been applied. Based on the paradigm, the corresponding ERP components including the motion-onset visual evoked potential N200 and P300 components were elicited by the stimuli as reported previously [23, 37]. Compared to the traditional P300-based BCI system, the increased ERP component N200 may help to improve the classification accuracy of the BCI. From the results of healthy subjects, the negative component (between 200 and 250 ms) of the specific N200 was found in the temporo-occipital and associated parietal cortical areas, including O1, Oz, and Pz. A clear P300 component emerged in the central and parietal regions, including Cz and Pz. Furthermore, a positive component after 500 ms that resembled the late positive component was exhibited in O1 and O2. There was no negative component in the ERP waveforms of the patients in the responsive group, but a broad P300 component was evident in each patient’s waveform. The differences in ERP waveforms between the healthy participants and DOC patients might be due to the severe brain injuries and limited cognitive levels of these patients.

In summary, among the 15 patients, two (patients 11 and 14) showed visual pursuit (higher visual function) or cognitive behavior in CRS-R assessment, but visual fixation was not detected by the BCI system. Actually, this phenomenon has been discussed in previous studies of visual fixation assessment [6]. According to the guidelines for assessing visual fixation in the CRS-R (e.g., use an object), many DOC patients fail to show visual fixating behavior while higher cognitive functions are present [6]. Our BCI system simulated the standard protocol in CRS-R, using a brightly-colored ball as the visual stimulus. Therefore, the phenomenon also occurred in our BCI system for visual fixation assessment; changing the visual stimulus described in the standard protocol (i.e., a colored ball or a bright light) may improve the detection of visual fixation such that the accuracy of BCI assessment is also improved. More importantly, one patient who did not show visual fixation behavior was judged to be visual fixation responsive by the BCI assessment. This result indicated that the BCI method may assist the clinical assessment of visual fixation in patients with DOCs and alleviate the risk of misdiagnosis.

The proposed BCI system aimed to complement the classical behavioral assessment for DOC patients. Therefore, the score on the visual fixation item should be revised according to the results of CRS-R and BCI. (1) If the DOC patient obtains consistent results (positive or negative) from both CRS-R and BCI, the item score of visual fixation remains unchanged. Specifically, if a reliable and consistent visual fixation behavior is detected by CRS-R and the accuracy of BCI is higher than the significance level, the score of visual fixation is 2. Conversely, if the results from both assessments are negative, the patient lacks visual fixation behavior or both methods missed it. The score is < 2 and may depend on assessment of the lower item (e.g., visual startle) than the visual fixation. (2) If a repeatable visual fixation behavior is detected by CRS-R but the result of the BCI is negative (missed), then the CRS-R result is preferred because a proportion of people are BCI illiteracy, which is that BCI control does not work for a few of users. (3) If visual fixation behavior is not observed in the patient by CRS-R but a significant accuracy rate in the BCI test is achieved, then the BCI result has priority and the score of visual fixation item is revised to 2.

This pilot study proposed a novel BCI system that provides assessment of visual fixation in DOC patients. In contrast to previous studies of BCI detection of consciousness, which still requires high-level cognitive abilities, we designed a BCI system simulating the visual fixation behavioral assessment. Combining the CRS-R- and BCI-based assessments may provide a degree of correction for the behavioral evaluation. In future, some improvements can be made. First, the averaged online accuracy of healthy subjects was 84% ± 3.4%, while the highest online accuracy of responsive patients judged by the BCI system only achieved 65%. Four responsive patients in BCI assessment had specific ERP patterns and showed different temporal properties. Furthermore, we used no special method for reducing artifacts in this study, as in many studies. Future investigators may consider how to design a feature-selection algorithm (e.g., channel selection) to be adapted to these patients for improving the detection performance of our BCI system and the preprocessing method can be elaborated to reduce artifacts in the EEG signals. Second, increasing the number of participants is underway for improving the feasibility of our system in clinical applications. Third, developing an integrated BCI system based on several assessment items of the CRS-R from our previous work is our goal in the future.
